# Associations between IL-1β, IL-6, and TNFα polymorphisms and longitudinal trajectories of cognitive function in non-demented older adults

**DOI:** 10.1016/j.bbih.2024.100816

**Published:** 2024-06-29

**Authors:** Karen A. Lawrence, Elana M. Gloger, Cristina N. Pinheiro, Frederick A. Schmitt, Suzanne C. Segerstrom

**Affiliations:** a College of Social Work, University of Kentucky, Lexington, KY, United States; b Department of Psychology, College of Arts and Sciences, University of Kentucky, Lexington, KY, United States; c Department of Neurology, College of Medicine, University of Kentucky, Lexington, KY, United States; d Sanders-Brown Center on Aging/Alzheimer's Disease Research Center, University of Kentucky, Lexington, KY, United States; e School of Human Development and Family Studies, Oregon State University, Corvallis, OR, United States

**Keywords:** Aged, Cytokines, Inflammation, Attention, Executive function, Polymorphism, Single nucleotide

## Abstract

Inflammation is implicated in Alzheimer's disease (AD), and specific single nucleotide polymorphisms (SNPs) in inflammatory cytokine genes are associated with increased AD risk. Whether the same polymorphisms also predict domain-specific cognitive change in cognitively healthy older adults is unclear. Specific SNPs in three cytokine genes, IL-1β (rs16944), IL-6 (rs1800795), and TNFα (rs1800629) were assessed for association with longitudinal trajectories spanning up to 16 years of global cognitive function, episodic memory, attention and working memory, and executive function in a sample of 324 non-demented older adults. Only rs1800629 (TNFα) was associated with significant change in global cognitive function over time [γ = 5.22; 95% CI: 0.61, 9.83; p = 0.027]. Despite an association with AD risk, rs16944 and rs1800795 may not predict cognitive decline in cognitively healthy older adults. The presence of an A at rs1800629 (TNFα) may have broad, protective effects on cognitive function, over time. More validation studies are needed to determine whether specific cytokine SNPs are associated with respective serum levels to further understanding of AD biomarkers that may also serve as markers of cognitive decline.

## 1 Introduction

Elevations in proinflammatory cytokine levels (e.g., IL-1β, IL-6, and TNFα) are implicated in the development of Alzheimer's Disease [AD] (Tan et al., 2007). IL-1β, IL-6, and TNFα are considered markers of neuroinflammation due to their presence in higher to lower graded concentrations in AD, MCI, and control patients, respectively (Leonardo and Fregni, 2023). In addition, recent meta-analyses found relationships between higher baseline IL-6 but not TNFα and steeper cognitive decline, with less published research on IL-1β levels and longitudinal cognitive change (Feng et al., 2023; Leonardo and Fregni, 2023). At the domain level, higher serum IL-6 is associated with longitudinal declines in psychomotor speed (Palta et al., 2015), semantic fluency (Lekander et al., 2011) and memory (Lekander et al., 2011; Schram et al., 2007) orientation and immediate word recall (Jordanova et al., 2007) processing speed, verbal fluency, non-verbal reasoning, and global cognitive function (Rafnsson et al., 2007). Although studies of associations between IL-1β levels and longitudinal domain-specific change are rare in healthy older adults, one prospective cohort study of European women (aged 45–90) reported that higher circulating IL-1β levels predicted better episodic recall and prospective memory (Lekander et al., 2011). We are not aware of similar domain-specific studies on TNFα. Therefore, the presence of only a few studies indicates that more studies are needed on risk factors for domain-specific cognitive decline, including risk factors associated with systemic proinflammatory cytokines.

### 1.1 SNPs and AD risk

Multiple studies reported associations between single nucleotide polymorphisms (SNPs) in IL-1β [rs16944] (Yucesoy et al., 2006), IL-6 [rs1800795], and TNFα [rs1800629] (Babić Leko et al., 2020) genes and AD risk although findings have been mixed (Mun et al., 2016; Ramos Dos Santos et al., 2016). A study of 241 Brazilians (Ramos Dos Santos et al., 2016) and a meta-analysis representative of Caucasians and Asians (Mun et al., 2016) found no association between AD and GC at rs1800795 (IL-6). A meta-analysis reported an association between CC at rs1800795 (IL-6) and a 30% lower AD risk compared to CG or GG (Dai et al., 2012). However, a case-control study of 943 Japanese American men, reported an association between TT at rs16944 (IL-1β) and increased AD and vascular dementia risk (Yucesoy et al., 2006). A case-control study of 116 Slovenian patients found an association between a C at rs16944 (IL-1β) and a 68.5% lower AD risk (Vogrinc et al., 2023). Finally, a case-control study of 1324 Finnish patients reported that an A at rs1800629 (TNFα) protected against AD (Sarajarvi et al., 2010).

At the domain level, associations between specific SNPs in IL-1β, IL-6, and TNFα and longitudinal trajectories of cognitive function have rarely been explored in non-demented older adults. Yet, if individual SNPs are reliably associated with systemic cytokine levels and longitudinal domain-specific cognitive change in domains (e.g., episodic memory, working memory, or executive function) associated with preclinical AD (Schindler et al., 2017; Wilson et al., 2011), mild cognitive impairment (MCI) (Grundman et al., 2004; Mistridis et al., 2015), and risk for dementia (Campbell et al., 2013; Mitchell and Shiri-Feshki, 2008; Petersen et al., 1999, 2009; Yaffe et al., 2006), they could serve as early biomarkers of cognitive change leading to preclinical AD and risk for MCI and dementia.

### 1.2 SNPs and cognitive function

CC at rs16944 (IL-1β) correlated with better working memory and abstraction/judgement performance in a cross-sectional study of 161 Chinese men, aged 69–92 years (Tsai et al., 2010). Better processing speed correlated cross-sectionally with A or AA at rs1800629 (TNFα) in a study of 369 community dwelling older adults (Baune et al., 2008). A cross-sectional study of 5653 older adults reported a small but significant correlation between CC at rs1800795 (IL-6) and worse selective attention; higher serum IL-6 correlated with worse cognitive performance yet rs1800795 did not significantly correlate with serum IL-6 (Mooijaart et al., 2013).

We aimed to determine whether cytokine SNPs linked to AD risk/protection are also associated with risk for or protection against cognitive decline. Clinically, such associations could yield early indicators of cognitive decline, detectable by genetic screening, and enabling timely interventions or preventive measures. We hypothesize that presence of an A allele at −511 (IL-1β promoter; rs16944) or a C allele at −174 (IL-6 promoter; rs1800795) is associated with domain-specific cognitive decline whereas an A allele at −308 in the TNFα promoter (rs1800629) is protective against decline in global function, episodic memory, attention/working memory, and executive function in the same non-demented older adults.

## 2 Materials and methods

### 2.1 Participants and procedures

De-identified data for secondary analyses were obtained from the University of Kentucky Alzheimer's Disease Research Center (UK-ADRC). Study approval was obtained for the parent study in which mental status and cognition were assessed annually; details of recruitment and inclusion criteria have been published (Schmitt et al., 2012). UK-ADRC genotype calls were downloaded directly from the National Alzheimer's Coordinating Center (NACC). DNA samples from research volunteers were provided to the National Centralized Repository for Alzheimer's Disease and Related Dementias (NCRAD) and processed by the Alzheimer's Disease Genetics Consortium (ADGC). Details on the ADGC's ADC genotyping waves are available in Bellenguez et al. (2022); Kunkle et al. (2019).

Of 324 participants (M_age_ = 72.8 years at baseline, *SD* = 6.09, range 57–92; 62.3% female, 93.8% white, M_edu_ = 16.7 years of education), 34% had at least one *APOE* Ɛ4 allele, 54% carried the A effect allele at rs16944, 67.6% carried the C allele at rs1800795, and 29.6% carried the A allele at rs1800629.

Of 935 possible participants, exclusion criteria were: <3 assessments (n = 201); refusal/problem/dementia too severe (n = 4); no genetic data (n = 446); psychiatric disorder, stroke history, Parkinson's, Down's syndrome, or Huntington's disease (n = 5); baseline mild cognitive impairment (MCI) or dementia diagnosis (n = 66); and incomplete data (n = 1). Participants completed 9 assessments on average (range = 3 to 16). Development of MCI or dementia after baseline (n = 118; 36.3%) was included as a covariate in adjusted models. Reasons for missingness at follow up included UK-ADRC discontinuation decision based on protocol (n = 6), participant refusal (n = 22), and 113 were deceased.

### 2.2 Measures and data analysis

Domain-specific cognitive scores were calculated using a regression-based, normative score calculator (Shirk et al., 2011): episodic memory [Logical Memory (Wechsler, 1987) and Craft Story 21 (Craft et al., 1996)]; attention/working memory [Digit Span Forward (Wechsler, 1987); number span forward (Weintraub et al., 2018)]; and executive function [animal and vegetable naming for category fluency (Morris et al., 1989) and the Trail Making Test – B (Reitan and Wolfsan, 1985)]. Global cognition was operationalized as weighted mean Mini Mental State Examination (MMSE) scores. The UK-ADRC replaced the MMSE with the Montreal Cognitive Assessment (MoCA) in 2015 (Monsell et al., 2016). MoCA scores from data collection preceding 2015 were converted to weighted mean MMSE scores according to Fasnacht et al. (2023) and were square transformed for normality. All other cognitive tests were standardized.

We used multilevel mixed models with time at Level 1 and people at Level 2 using PROC MIXED (SAS 9.4; SAS Institute, Cary, NC) with restricted maximum likelihood estimation and Kenward-Rogers degrees of freedom. Planned analyses were interactions of Level 2 covariates with time in the presence of genotype*time. These equations illustrate the multilevel model:

Level 1: Y_ij_ = β_0j_ + β_1j_(year) + e_ij_

Level 2: β_0j_ = γ_00_ + γ_01_(genotype) + U_0j_

Level 2: β_1j_ = γ_10_ + γ_11_(genotype) + U_1j_

For person j in year i, the outcome Y_ij_ was a function of time at year i and of between-person genotype j. Models included a random effect of intercept (U_0j_) and testing indicated significant model improvement with a random effect of year (Χ^2^ = 23.7, *p* < 0.0001) so year was included as a random effect (U_1j_) in all models. Slopes were examined after adjustment for baseline age, sex, education, MCI or dementia diagnosis during study (yes/no), and *APOE* Ɛ4 status (absent/present). Fixed effects are reported as unstandardized γ weights, with 95% confidence intervals. Separate models tested main effects of each SNP genotype coded as presence of A at rs16944; C at rs1800795; or A at rs1800629. Exploratory analyses examined the three-way interaction between the SNPs, *APOE* Ɛ4, and time, including all lower-level interactions (Table 1). A sensitivity analysis using 500 simulations indicated power >0.99 for the interaction for all tested combinations of random effect and interaction effect sizes (**supplementary method 1**).

**Table 1:** Effects of SNP alleles on cognition (N = 324).

rs16944																
	Global cognition				Episodic memory				Working memory				Executive function			
	Estimate (SE)	*p*	CI_low_	CI_high_	Estimate (SE)	*p*	CI_low_	CI_high_	Estimate (SE)	*p*	CI_low_	CI_high_	Estimate (SE)	*p*	CI_low_	CI_high_
Fixed Effects																
Intercept	**851.18 (19.44)**	**<**0**.0001**	812.95	889.41	0.61 (0.32)	0.06	−0.030	1.25	0**.94 (**0**.31)**	0**.003**	0.33	1.55	−0.15 (0.59)	0.80	−1.30	1.01
Time	**−6.47 (1.59)**	**<**0**.0001**	−9.60	−3.34	**−**0**.05 (**0**.01)**	**<**0**.0001**	−0.07	−0.02	**−**0**.07 (**0**.01)**	**<**0**.0001**	−0.08	−0.05	**−**0**.13 (**0**.02)**	**<**0**.0001**	−0.17	−0.09
rs16944 (Ref = Not present)	−14.6 (19.14)	0.111	−32.59	3.39	−0.06 (0.14)	0.66	−0.33	0.21	−0.10 (0.11)	0.39	−0.31	0.12	−0.02 (0.22)	0.91	−0.46	0.41
Baseline age	**−1.64 (**0**.45)**	0**.0003**	−2.52	−0.752	**−**0**.02 (**0**.01)**	0**.002**	−0.04	−0.01	−0.01 (0.01)	0.15	−0.02	0.00	**−**0**.06 (**0**.01)**	**<**0**.0001**	−0.09	−0.03
*APOE* Ɛ4 (Ref = Not Present)	9.25 (11.48)	0.42	−13.35	31.85	0.07 (0.17)	0.66	−0.26	0.41	−0.11 (0.14)	0.42	−0.39	0.16	0**.58 (**0**.28)**	0**.04**	0.03	1.12
Sex (Ref = Male)	**13.24 (5.50)**	0**.016**	2.42	24.05	0.14 (0.09)	0.12	−0.03	0.31	0.09 (0.09)	0.32	−0.08	0.26	0**.65 (**0**.16)**	**<**0**.0001**	0.33	0.97
Education	**3.08 (**0**.97)**	0**.002**	1.18	4.98	0.02 (0.02)	0.18	−0.01	0.05	−0.02 (0.02)	0.30	−0.05	0.01	0**.12 (**0**.03)**	**<**0**.0001**	0.07	0.18
Dementia/MCI during study period	**−31.039 (5.60)**	**<**0**.0001**	−42.06	−20.02	**−**0**.58 (**0**.09)**	**<**0**.0001**	−0.75	−0.40	−0.08 (0.09)	0.34	−0.26	0.09	**−1.00 (**0**.17)**	**<**0**.0001**	−1.3	−0.67
rs16944 *Time	−0.950 (2.14)	0.66	−5.17	3.27	−0.01 (0.02)	0.63	−0.04	0.03	0.00 (0.01)	0.70	−0.02	0.03	−0.01 (0.03)	0.71	−0.07	0.05
rs16944 *APOE Ɛ4	5.72 (15.70)	0.72	−25.19	36.62	−0.14 (0.23)	0.58	−0.60	0.32	0.20 (0.19)	0.29	−0.17	0.58	0.14 (0.38)	0.71	−0.60	0.88
*APOE* Ɛ4 *Time	**−5.53 (2.69)**	0**.04**	−10.82	−0.23	−0.03 (0.02)	0.13	−0.08	0.01	−0.02 (0.01)	0.27	−0.04	0.01	**−**0**.13 (**0**.04)**	0**.0003**	−0.20	−0.06
rs16944 *Time *APOE Ɛ4	2.91 (3.69)	0.43	−4.35	10.18	0.04 (0.03)	0.20	−0.02	0.10	0.03 (0.02)	0.18	−0.01	0.06	0.02 (0.05)	0.62	−0.07	0.12
Random effects																
Level 2 variance	**2288.43**				0**.765**				0**.499**				**1.896**			
Slope variance	**164.24**				0**.010**				0**.002**				0**.022**			
Level 2 – slope variance	**−396.54**				**−**0**.054**				−0.006				**−**0**.070**			
Level 1 variance	**4367.94**				0**.479**				0**.358**				**1.439**			

**rs1800795**																
	Global cognition				Episodic memory				Working memory				Executive function			
	Estimate (SE)	*p*	CI_low_	CI_high_	Estimate (SE)	*p*	CI_low_	CI_high_	Estimate (SE)	*p*	CI_low_	CI_high_	Estimate (SE)	*p*	CI_low_	CI_high_
Fixed Effects																
Intercept	**844.67 (20.02)**	**<**0**.0001**	805.30	884.04	0.58 (0.33)	0.08	−0.06	1.23	0**.78 (**0**.31)**	0**.01**	0.16	1.40	−0.35 (0.59)	0.55	−1.5	0.81
Time	**−8.39 (1.88)**	**<**0**.0001**	−12.09	−4.69	**−**0**.05 (**0**.02)**	0**.002**	−0.08	−0.02	**−**0**.06 (**0**.01)**	**<**0**.0001**	−0.08	−0.04	**−**0**.14 (**0**.03)**	**<**0**.0001**	−0.19	−0.09
rs1800795 (Ref = Not present)	−3.57 (9.74)	0.71	−22.75	15.61	0.03 (0.15)	0.85	−0.26	0.32	0.16 (0.12)	0.18	−0.08	0.39	0.44 (0.23)	0.06	−0.02	0.90
Baseline age	**−1.58 (**0**.46)**	0**.0006**	−2.48	−0.68	**−**0**.02 (**0**.01)**	0**.002**	−0.04	−0.01	−0.01 (0.01)	0.15	−0.02	0.00	**−**0**.06 (**0**.01)**	**<**0**.0001**	−0.09	−0.04
*APOE* Ɛ4 (Ref = Not Present)	25.06 (13.66)	0.068	−1.83	51.96	−0.07 (0.20)	0.74	−0.47	0.34	0.06 (0.17)	0.73	−0.27	0.39	0**.74 (**0**.33)**	0**.02**	0.10	1.39
Sex (Ref = Male)	**14.55 (5.55)**	0**.009**	3.62	25.48	0.14 (0.09)	0.11	−0.03	0.31	0.09 (0.09)	0.30	−0.08	0.26	0**.64 (**0**.16)**	**<**0**.0001**	0.32	0.95
Education	**3.02 (**0**.98)**	0**.002**	1.10	4.95	0.02 (0.02)	0.20	−0.01	0.05	−0.02 (0.02)	0.32	−0.05	0.01	0**.12 (**0**.03)**	**<**0**.0001**	0.07	0.18
Dementia/MCI during study period	**−31.13 (5.66)**	**<**0**.0001**	−42.27	−20.00	**−5.8 (**0**.09)**	**<**0**.0001**	−0.75	−0.40	−0.09 (0.09)	0.29	−0.27	0.08	**−1.01 (**0**.16)**	**<**0**.0001**	−1.34	−0.69
rs1800795 *Time	2.00 (2.28)	0.38	−2.48	6.48	0.00 (0.02)	0.92	−0.04	0.40	−0.01 (0.01)	0.59	−0.03	0.02	0.02 (0.03)	0.58	−0.04	0.08
rs1800795 *APOE Ɛ4	−18.35 (16.60)	0.27	−51.03	14.34	0.12 (0.25)	0.64	−0.37	0.61	−0.10 (0.20)	0.63	−0.49	0.30	−0.13 (0.40)	0.75	−0.91	0.65
*APOE* Ɛ4 *Time	**−6.60 (3.18)**	0**.039**	−12.87	−0.33	−0.02 (0.03)	0.38	−0.08	0.03	0.00 (02)	0.77	−0.03	0.04	**−**0**.14 (**0**.04)**	0**.001**	−0.22	−0.05
rs1800795 *Time *APOE Ɛ4	4.04 (3.89)	0.30	−3.61	11.70	0.02 (0.03)	0.64	−0.05	0.08	−0.00 (0.02)	0.63	−0.05	0.03	0.03 (0.05)	0.55	−0.07	0.13
Random effects																
Level 2 variance	**2278.41**				0**.767**				0**.498**				**1.85**			
Slope variance	**160.88**				0**.010**				0.002				0**.021**			
Level 2 – slope variance	**−381.38**				**−**0**.054**				**−**0**.005**				**−**0**.07**			
Level 1 variance	**4368.51**				0**.479**				0**.359**				**1.44**			

**rs1800629**																
	Global cognition				Episodic memory				Working memory				Executive function			
	Estimate (SE)	*p*	CI_low_	CI_high_	Estimate (SE)	*p*	CI_low_	CI_high_	Estimate (SE)	*p*	CI_low_	CI_high_	Estimate (SE)	*p*	CI_low_	CI_high_
Fixed Effects																
Intercept	**849.07 (19.25)**	**<**0**.0001**	811.19	886.94	0.61 (0.32)	0.06	−0.02	1.24	0**.96 (**0**.31)**	0**.002**	0.35	1.56	−0.06 (0.58)	0.92	−1.20	1.09
Time	**−8.53 (1.25)**	**<**0**.0001**	−10.99	−6.07	**−**0**.06 (**0**.02)**	**<**0**.0001**	−0.08	−0.03	**−**0**.07 (**0**.006)**	**<**0**.0001**	−0.08	−0.06	**−**0**.14 (**0**.02)**	**<**0**.0001**	−0.17	−0.11
rs1800629 (Ref = Not present)	**−22.19 (10.04)**	0**.027**	−41.95	−2.42	−0.09 (0.15)	0.56	−0.39	0.21	−0.24 (0.12)	0.05	−0.48	0.00	−0.22 (0.24)	0.36	−0.70	0.26
Baseline age	**−1.62 (**0**.46)**	0**.0004**	−2.53	−0.72	**−**0**.02 (**0**.01)**	0**.002**	−0.04	−0.01	−0.01 (0.01)	0.11	−0.03	0.00	**−**0**.06 (**0**.01)**	**<**0**.0001**	−0.09	−0.04
*APOE* Ɛ4 (Ref = Not Present)	8.76 (9.52)	0.36	−9.99	27.50	−0.08 (0.14)	0.56	−0.37	0.20	−0.10 (0.12)	0.39	−0.33	0.13	0**.50 (**0**.23)**	0**.03**	0.04	0.96
Sex (Ref = Male)	**14.13 (5.56)**	0**.012**	3.19	25.07	0.15 (0.09)	0.09	−0.02	0.32	0.09 (0.09)	0.32	−0.08	0.25	0**.65 (**0**.16)**	**<**0**.0001**	0.33	0.97
Education	**3.04 (**0**.98)**	0**.002**	1.12	4.97	0.02 (0.02)	0.19	−0.01	0.05	−0.01 (0.02)	0.35	−0.04	0.02	0**.12 (**0**.03)**	**<**0**.0001**	0.07	0.18
Dementia/MCI during study period	**−30.59 (5.67)**	**<**0**.0001**	−41.74	−19.44	**−**0**.58 (**0**.09)**	**<**0**.0001**	−0.76	−0.40	−0.09 (0.09)	0.33	−0.26	0.09	**−1.01 (**0**.17)**	**<**0**.0001**	−1.33	−0.68
rs1800629 *Time	**5.22 (2.34)**	0**.027**	0.61	9.83	0.02 (0.02)	0.36	−0.02	0.06	0.02 (0.01)	0.13	−0.01	0.04	0.03 (0.03)	0.42	−0.04	0.09
rs1800629 *APOE Ɛ4	15.11 (16.90)	0.37	−18.18	48.39	0.29 (0.25)	0.25	−0.21	0.80	0.30 (0.21)	0.15	−0.11	0.70	0.47 (0.41)	0.25	−0.33	1.28
*APOE* Ɛ4 *Time	−3.40 (2.19)	0.12	−7.71	0.92	−0.00 (0.02)	0.82	−0.04	0.03	0.00 (0.01)	0.74	−0.02	0.03	**−**0**.12 (**0**.03)**	**<**0**.0001**	−0.18	−0.06
rs1800629 *Time *APOE Ɛ4	−2.27 (3.96)	0.57	−10.07	5.54	−0.03 (0.03)	0.38	−0.10	0.04	−0.02 (0.02)	0.37	−0.06	0.02	−0.01 (0.05)	0.86	−0.12	0.10
Random effects																
Level 2 variance	**2231.22**				0**.765**				0**.494**				**1.89**			
Slope variance	**160.38**				0**.010**				0.002				0**.022**			
Level 2 – slope variance	**−372.68**				**−**0**.054**				**−**0**.005**				**−**0**.069**			
Level 1 variance	**4366.37**				0**.479**				0**.359**				**1.44**			

Bold = *p* < 0.05; * indicates interaction.

## 3 Results

Only the presence of C at rs1800795 (IL-6) correlated significantly with better executive function, on average (r = 0.17, p = 0.002; Supplementary Table 1). Of the three SNPs tested, the only significant relationship with cognitive function over time was rs1800629 (TNFα) which was associated with positive global cognitive function slopes in adjusted models such that those with the effect allele showed a 5.22 squared unit increase in global function, per assessment [γ = 5.22; 95% CI: 0.61, 9.83; p = 0.027] (Table 1). This translates to an increase of 2.29 points in MMSE score per year.

There were no significant SNP*APOE Ɛ4 nor SNP*APOE Ɛ4*time interaction effects regardless of adjustment for covariates (Table 1). As expected, development of MCI or dementia was significantly negatively correlated with scores across each cognitive domain (Supplementary Table 1) and more negative slopes were associated with *APOE* Ɛ4 genotype (Table 1).

We conducted post-hoc analyses to test for associations between SNPs and plasma biomarker levels in this sample, the presumed mechanism for effects of the SNPs. However, variability between and within the assays precluded confidence in the results (Estepp et al., 2023).

## 4 Discussion

Although these SNPs have been associated with risk for or protection against AD, only rs1800629 (TNFα) predicted cognitive change in cognitively healthy older adults. This was the first study, to our knowledge, to examine the relationship between rs16944 (IL-1β) and domain-specific cognitive trajectories and our findings indicate no relationship. Notably, higher IL-1β levels were associated with a GA at −511 [rs16944] in Mexican patients with antisynthetase syndrome (Ponce-Gallegos et al., 2020) suggesting a correspondence between the SNP and serum IL-1β levels but SNP - IL-1β validation studies in adults are rare.

To our knowledge, no prior study has examined rs1800795 (IL-6) effects on domain-specific cognitive trajectories. Despite the association of IL-6 levels with increased risk of cognitive decline (Feng et al., 2023; Leonardo and Fregni, 2023), our findings indicate no relationship between rs1800795 and longitudinal cognitive change in the domains tested, in healthy older adults. Interestingly, one cross-sectional study noted an association between IL-6 plasma levels and worse selective attention yet rs1800795 was not associated with IL-6 levels (Mooijaart et al., 2013). It may be that there are cell type-specific gene expression patterns influenced by rs1800795 yet these would not be detected in circulating IL-6. For example, one study found an association between CC at rs1800795 and IL-6 levels in synovial fibroblasts but not monocytes (Noss et al., 2015). Future research could test whether other SNPs in the IL-6 gene region regulate IL-6 expression in peripheral blood cells and potential associations with cognitive decline.

As expected, A or AA at rs1800629 (TNFα), which a prior study showed was associated with elevated TNFα levels (Pan et al., 2019) was not associated with decline in any domain tested. Rather, individuals with the effect allele showed a 2.33 increase in global cognitive functioning (MMSE) score, annually, relative to those without the effect allele. This is a clinically meaningful change based on norms for reliable change in cognitively normal older adults (Hensel et al., 2007). Although higher TNFα is associated with AD pathology (Babić Leko et al., 2020; Perry et al., 2001), peripheral TNFα level may not be a good indicator of neurodegeneration (Feng et al., 2023; Leonardo and Fregni, 2023). For example, findings from Feng and colleagues’ (2023) meta-analysis suggested that older adults with higher peripheral CRP, IL-6, and TNFα levels had a 14% increased risk of future cognitive decline yet in the subgroup analysis of cytokine types, cognitive decline was significantly correlated with increased IL-6 but not CRP or TNFα levels. Our results are in accord with this based on an assumption that A or AA at rs1800629 does indeed correspond to higher circulating TNFα levels. Elevated serum TNFα was associated with AA and AG genotypes at −308 [rs1800629] in Iraqi (Ahmed et al., 2020) and Chinese patients (Pan et al., 2019) with vitiligo and congenital heart disease, respectively. Yet, no baseline association between rs1800629 and TNFα serum levels was observed in Slovenian patients with coronary artery disease (Levstek et al., 2022) suggesting a need for more research to clarify this relationship.

Study findings should be considered in the context of the limitations including low racial and ethnic diversity in the study sample potentially limiting generalizability to non-white and Hispanic populations. Another limitation is that the SNPs examined could not be validated for correspondence with concentration in blood samples. Strengths of the study are the longitudinal design as most prior studies on genotype associations with cognitive functions have been cross-sectional. Additionally, the present study findings were free of potential confounding by psychiatric and neurodegenerative conditions.

## 5 Conclusion

In conclusion, rs1800629 (TNFα) was associated with a maintenance of global cognitive function, presumably due to broad neuroprotective effects yet validation studies to confirm an association with TNFα concentration in peripheral blood are needed. Future studies could examine if SNPs other than rs1800795 in the IL-6 gene region are associated with circulating IL-6 levels. In general, more validation studies on cytokine SNP associations with serum or plasma levels of the respective protein are needed for potential identification of early biomarkers of cognitive change that could lead to preclinical AD and risk for MCI and dementia.

## Funding

This work was funded by K01AG070279 awarded to K.A. Lawrence and P30 AG072946 awarded to the 10.13039/100007472University of Kentucky Alzheimer's Disease Research Center from the 10.13039/100000049National Institute on Aging (10.13039/100000049NIA) at the 10.13039/100000002National Institutes of Health (10.13039/100000002NIH). This content is solely the responsibility of the authors and does not necessarily represent the official views of the NIH's NIA.

## CRediT authorship contribution statement

**Karen A. Lawrence:** Writing – review & editing, Writing – original draft, Methodology, Funding acquisition, Data curation, Conceptualization. **Elana M. Gloger:** Formal analysis, Data curation. **Cristina N. Pinheiro:** Writing – review & editing. **Frederick A. Schmitt:** Supervision, Methodology, Funding acquisition, Conceptualization. **Suzanne C. Segerstrom:** Writing – review & editing, Supervision, Methodology, Formal analysis, Conceptualization.

## Declaration of competing interest

none.

## Data Availability

Data will be made available on request.
