# Supplementary material for: Associations between IL-1β, IL-6, and TNFα polymorphisms and longitudinal trajectories of cognitive function in non-demented older adults
Source: Brain Behav Immun Health. 2024 Jun 29;39:100816. doi: 10.1016/j.bbih.2024.100816 (PMC11269286; doi:10.1016/j.bbih.2024.100816)
Supplement: Multimedia component 1 [file mmc1.docx]

**Supplementary Method 1**

A sensitivity analysis was performed using the R package *paramtest* (Hughes, 2017). For each combination of interaction effect size and intraclass correlation (ICC) as detailed below, 500 simulations were performed and power for α = .05 and N = 324 (sample N) was calculated. The simulated model was:

Level 1: Y_ij_ = β_0j_ + β_1j_(year_ij_) + e_ij_

Cognitive function for person j at time i is a function of cognitive function at the first assessment (intercept β_0j_), time (β_1j_), and residual variance (e_ij_)

Level 2: β_0j_ = γ_00_ + γ_01_(genotype) + U_0j_

β_1j_ = γ_10_ + γ_11_(genotype) + U_1j_

The intercepts are a function of a sample intercept (γ_00_), the effect of a SNP (γ_01_), and residual Level 2 intercept variance (random intercepts U_0j_).

The time slopes are a function of a sample slope (γ_10_), the effect of a SNP (γ_11_), and residual Level 2 slope variance (random time slopes U_1j_).

Model parameters were defined as follows with their rationale.

Data

year = 0 - 8 Number of assessments (sequential)

genotype = 0, 1 Drawn from binomial distribution with p = .703 (most unbalanced in data)

Fixed effects

γ_00_ = 0 Intercept

γ_01_ = .20 Small to medium main effect of genotype

γ_10_ = -0.15 Larger main effect of time than across the entire lifespan (Tucker-Drob et al., 2019), but slope of decline is larger with increasing age (Wilson et al., 2002).

γ_11_ = 0.1, 0.2, 0.3 Range of time*genotype interaction effect sizes from small to medium

Random effects

Var(U_0j_) = .41, .55 Drawn from normal distribution with variance equal to the lowest or highest ICCs among the study outcomes

Var(U_1j_) = Var(U_0j_)*.03 Drawn from normal distribution with variance equal to 3% of the intercept variance (magnitude of slope variance ranged from 0.35% to 10.5% of the intercept variance in the study models)

Var(e_ij_) 1 - lowest and highest ICC among the study outcomes

The key outcome was power to detect a significant interaction between genotype and time (γ_11_) across different fixed effect sizes and random effect sizes. Results of the simulation indicated power > .99 for each combination.

| Effect size | ICC (random intercept) | Power |
| --- | --- | --- |
| 0.1 | 0.41 | > .99 |
| 0.1 | 0.55 | > .99 |
| 0.2 | 0.41 | > .99 |
| 0.2 | 0.55 | > .99 |
| 0.3 | 0.41 | > .99 |
| 0.3 | 0.55 | > .99 |

Hughes, J. (2017). *paramtest: Run a Function Iteratively While Varying Parameters*. https://CRAN.R-project.org/package=paramtest

Tucker-Drob, E. M., Brandmaier, A. M., & Lindenberger, U. (2019). Coupled cognitive changes in adulthood: A meta-analysis. *Psychological Bulletin*, *145*(3), 273–301. https://doi.org/10.1037/bul0000179

Wilson, R. S., Beckett, L. A., Barnes, L. L., Schneider, J. A., Bach, J., Evans, D. A., & Bennett, D. A. (2002). Individual differences in rates of change in cognitive abilities of older persons. *Psychology and Aging*, *17*(2), 179–193. https://doi.org/10.1037/0882-7974.17.2.179
