# Supplementary material for: Associations between IL-1β, IL-6, and TNFα polymorphisms and longitudinal trajectories of cognitive function in non-demented older adults
Source: Brain Behav Immun Health. 2024 Jun 29;39:100816. doi: 10.1016/j.bbih.2024.100816 (PMC11269286; doi:10.1016/j.bbih.2024.100816)
Supplement: Multimedia component 2 [file mmc2.docx]

Supplementary Table 1

*Descriptive Statistics*

| Variable | *M (SD)* | 1 | 2 | 3 | 4 | 5 | 6 | 7 | 8 | 9 | 10 | 11 | 12 | 13 |
| --- | --- | --- | --- | --- | --- | --- | --- | --- | --- | --- | --- | --- | --- | --- |
| 1. Age at 1^st^ assessment | 72.8 (6.09) | 1 |  |  |  |  |  |  |  |  |  |  |  |  |
| 2. Sex  (Female) | 62.3% | **-.16**  **[-.26, -.05]** | 2 |  |  |  |  |  |  |  |  |  |  |  |
| 3. Education | 16.7 (2.7) | .06  [-.05, .17] | **-.12**  **[-.23, -.02]** | 3 |  |  |  |  |  |  |  |  |  |  |
| 4. Race (Caucasian) | 93% | **-.11**  **[-.22, -.00]** | .**12**  **[.01, .23]** | **-.14**  **[-.24, -.03]** | 4 |  |  |  |  |  |  |  |  |  |
| 5. Total number of assessments | 9.8 (3.5) | [-.27, -.06] | .03  [-.08, .14] | -.05  [-.15, .06] | **-.20**  **[-.30, -.09]** | 5 |  |  |  |  |  |  |  |  |
| 6. Developed MCI or dementia | 36.4% | .**19**  **[.08, .29]** | **-.11**  **[-.22, -.00]** | -.02  [-.13, .09] | -.03  [-.14, .08] | **.14**  **[.03, .24]** | 6 |  |  |  |  |  |  |  |
| 7. *APOE* Ɛ4 status (% present) | 34% | **-.18**  **[-.28, -.07]** | .01  [-.10, .11] | .05  [-.06, .16] | -.08  [-.18, .03] | -.02  [-.13, .09] | **.13**  **[.03, .24]** | 7 |  |  |  |  |  |  |
| 8. Global Cognition | 28.7 (1.2) | **-.29**  **[-.38, -.18]** | **.14**  **[.03, .24]** | **.13**  **[.02, .24]** | **-.15**  **[-.25, -.04]** | **.14**  **[.03, .24]** | **-.54**  **[-.61, -.46]** | -.04  [-.14, .07] | 8 |  |  |  |  |  |
| 9. Episodic Memory |  | **-.23**  **[-.33, -.12]** | .**14**  **[.03, .25]** | .05  [-.06, .16] | **-.18**  **[-.28, -.07]** | **.18**  **[.07, .28]** | **-.44**  **[-.52, -.35]** | -.05  [-.16, .06] | .**57**  **[.49, .64]** | 9 |  |  |  |  |
| 10. Working Memory |  | -.08  [-.19, .03] | .06  [-.04, .17] | -.06  [-.17, .05] | .04  [-.07, .14] | **.13**  **[.02, .23]** | **-.12**  **[-.23, -.01]** | -.00  [-.11, .11] | **.17**  **[.06, .27]** | **.14**  **[.04, .25]** | 10 |  |  |  |
| 11. Executive Function |  | **-.28**  **[-.38, -.18]** | **.24**  **[.14, .34]** | **.18**  **[.07, .28]** | -.10  [-.21, .01] | .09  [-.02, .20] | **-.44**  **[-.53, -.35]** | .04  [-.07, .14] | **.52**  **[.44, .60]** | **.46**  **[.37, .54]** | **.15**  **[.04, .25]** | 11 |  |  |
| 12. rs16944  (A is effect allele; % present) | 46% | -.02  [-.13, .09] | -.02  [-.12, .09] | -.00  [-.11, .11] | **.17**  **[.06, .27]** | -.00  [-.11, .11] | -.02  [-.13, .09] | -.01  [-.12, .10] | -.07  [-.17, .04] | -.05  [-.16, .06] | .01  [-.10, .12] | .00  [-.11, .11] | 12 |  |
| 13. rs1800795 (C is effect allele; % present) | 32.4% | .09  [-.02, .20] | .01  [-.09, .12] | .02  [-.09, .13] | **-.23**  **[-.33, -.12]** | -.05  [-.15, .06] | .02  [-.09, .13] | -.05  [-.15, .06] | .05  [-.06, .15] | .04  [-.07, .15] | .04  [-.07, .14] | .**17**  **[.06, .27]** | -.06  [-.16, .05] | 13 |
| 14. rs1800629 (A is effect allele; % present) | 70.4% | -.04  [-.15, .07] | -.06  [-.17, .05] | .06  [-.05, .17] | -.04  [-.14, .07] | .01  [-.10, .12] | .01  [-.10, .12] | .06  [-.05, .17] | .06  [-.05, .17] | .03  [-.08, .14] | -.02  [-.12, .09] | .02  [-.09, .13] | -.07  [-.17, .04] | -.03  [-.14, .08] |

*Note.* Bold = *p* < .05; *M* = mean; *SD* = standard deviation; [ ] = 95% confidence interval for correlation.
